# A Trusted AI-Driven Parent-Baby Companion App as a Perioperative Support Tool for Pediatric Surgical Practice

**DOI:** 10.7759/cureus.113116

**Published:** 2026-07-21

**Authors:** Thom E Lobe

**Affiliations:** 1 Surgery, University of Illinois Chicago, Chicago, USA

**Keywords:** ai in pediatric surgery, artificial intelligence, caregiver support, digital health, pediatric surgery, perioperative care, postoperative monitoring

## Abstract

Pediatric surgical care increasingly relies on families to manage preparation, discharge instructions, symptom monitoring, hydration, pain control, and escalation decisions at home after brief encounters with the surgical team. A trusted, reliable, personalized AI-driven parent-baby companion app may help pediatric surgical practices extend perioperative support beyond the clinic or hospital by improving caregiver preparedness, standardizing education, reducing repetitive postoperative calls, structuring home-reported data, and supporting earlier recognition of complications. Existing pediatric digital health literature suggests that app-based preparation and postoperative monitoring can improve caregiver experience, support pain assessment, and reduce communication burden when tools are embedded into clinical workflows. This article outlines a practice-oriented framework for how such an app could support pediatric surgical care, with emphasis on calm parent guidance, procedure-specific recovery pathways, postoperative triage, and workflow integration. The app is best conceptualized as a conservative clinical support layer that reinforces surgeon-approved instructions rather than replacing clinician judgment.

## Introduction

Pediatric surgery is clinically intense but operationally brief. Families often spend limited time with the surgical team before they are expected to manage fasting instructions, medications, wound monitoring, fluid intake, pain control, and recovery decisions at home [1,2]. This creates a predictable gap between what the clinical team intends and what caregivers can confidently execute after discharge.

Parental anxiety is a major feature of this gap. Children scheduled for surgery and anesthesia commonly experience fear and distress, while parents face uncertainty about preparation, postoperative symptoms, and when to seek help [1,2]. In outpatient pathways, these concerns may drive repetitive after-hours calls, unnecessary urgent care use, delayed recognition of complications, and inconsistent adherence to discharge plans [1,2].

The growth of artificial intelligence (AI) and digital health in pediatric care has created an opportunity to address these issues through structured, personalized support tools [3,4]. The American Academy of Pediatrics has recognized that AI tools in pediatric health care may help decrease burden, promote equity, and improve care quality when implemented appropriately [3]. Within pediatric surgery, a practice-affiliated AI companion app could provide a continuous perioperative support layer that improves family guidance while making surgical follow-up more efficient and data-informed.

## Technical report

Rationale for use in pediatric surgical practice

A pediatric surgical practice has several characteristics that make it especially well suited for a companion app model. First, home recovery is often standardized enough to support structured guidance, yet variable enough to generate large numbers of caregiver questions [1,2]. Second, many common postoperative concerns, such as pain patterns, hydration, nausea, constipation, wound appearance, and activity restrictions, can be triaged more effectively when families report them in a structured format [4,5]. Third, the experience is highly emotional, which makes calm, trusted, always-available guidance especially valuable [1,2].

Michigan Medicine reported that, in a pilot involving children discharged after tonsil surgery, families using a smartphone application generated fewer clinic calls than those receiving standard paper instructions, and most families preferred the app format because it was easier to use and more helpful [2]. These findings suggest that a pediatric surgery-specific app may offer meaningful operational value when designed around procedure-specific recovery workflows.

Preoperative preparation

Preoperative preparation is a high-value starting point. In a randomized study of a mobile app intervention for preschool children undergoing day surgery, the app delivered information before and after surgery through a timeline with text, images, and videos and was designed to support both children and parents [1]. For a pediatric surgical practice, a similar tool could provide fasting reminders, medication holds, arrival instructions, consent preparation, equipment checklists, and age-matched explanations of the procedure [1].

**Figure 1:**
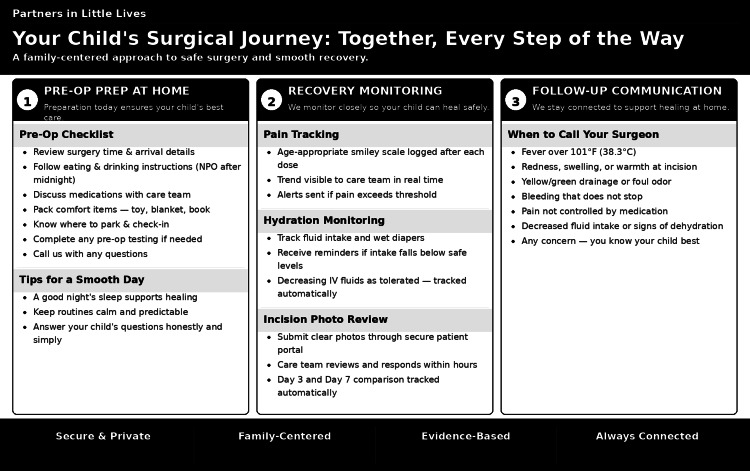
Perioperative Journey Perioperative journey support through a proposed pediatric surgical companion app. This schematic depicts three linked phases of support: preoperative preparation at home, postoperative monitoring after discharge, and follow-up communication with the surgical team. This schematic was created by the author using Python and the Pillow imaging library (Python Software Foundation; Pillow Contributors, https://python-pillow.org)

This function may reduce preventable delays and cancellations by improving adherence to preoperative instructions [1]. It also promotes standardization across surgeons, offices, and surgery centers while still allowing content to be personalized to procedure type, diagnosis, age, and comorbid conditions. A proposed perioperative journey illustration is shown in Figure 1.

Postoperative monitoring and symptom triage

Postoperative home monitoring is likely the area of greatest impact. AI applications in pediatric health management increasingly include longitudinal monitoring, communication support, predictive analytics, and clinical decision support [4]. For pediatric surgery, an app could prompt caregivers to document oral intake, emesis, urine output, pain scores, fever, stooling, wound findings, drain output, mobility, and sleep in a structured manner after discharge [4,5].

**Figure 2:**
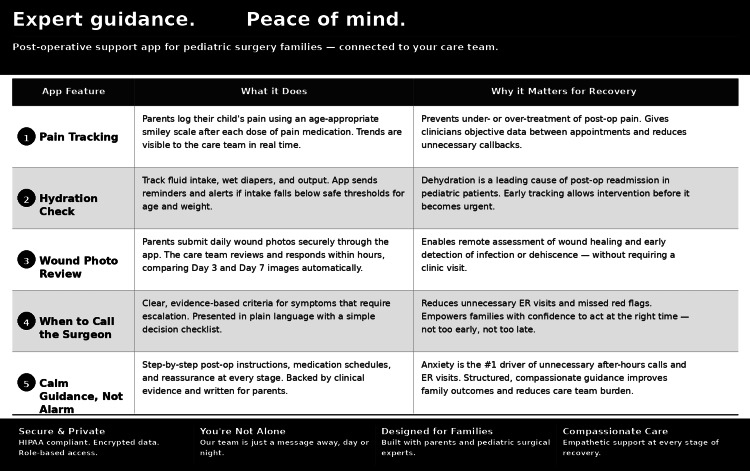
Postoperative Support Parent-facing postoperative support. This schematic depicts how a parent using smartphone-based companion app features after surgery might help. This schematic was created by the author using Python and the Pillow imaging library (Python Software Foundation; Pillow Contributors, https://python-pillow.org)

This structured monitoring changes the quality of communication. Rather than an unstructured message such as “my child is not doing well,” the surgical team can receive a summary that includes postoperative day, hydration status, analgesic timing, pain trend, and wound appearance. That type of report supports more accurate triage and earlier prioritization of high-risk patients [4]. A conceptual parent-facing postoperative support view is shown in Figure 2.

AI-based clinical decision support has potential advantages over traditional rule-based systems through individualized risk assessment and reduced false alerts in appropriate contexts [6]. The AI architecture envisioned for this application is intended to combine several complementary techniques, each addressing a distinct functional need. Natural language understanding of parent-submitted queries and generation of calm, reassurance-first, procedure-specific guidance in plain language would be handled by a large language model (LLM). To prevent hallucinated or ungrounded advice, the LLM's outputs are intended to be constrained through retrieval-augmented generation, in which responses are generated only from a curated, surgeon-approved knowledge base of procedure-specific instructions and escalation criteria, rather than from the model's unconstrained training data. Layered above this is a deterministic, rule-based escalation module that encodes fixed clinical thresholds, for example, fever above 101°F, new or worsening wound drainage, or absent urine output, which are designed to override any AI-generated response and trigger an immediate escalation pathway, functioning as a non-AI safety net independent of model behavior. Structured inputs such as pain scores, hydration logs, and wound photographs would additionally be processed through machine learning classifiers trained to recognize abnormal symptom-trend patterns and flag them for clinician review; a computer vision component may optionally be incorporated to analyze wound photographs for color change, dehiscence, or drainage. Consistent with a conservative design philosophy, the system is not intended to render autonomous diagnoses. Its proposed role is limited to urgency classification and routing of communication, with final clinical interpretation and decision-making remaining exclusively with the surgeon. In pediatric surgery, however, the safest model is conservative augmentation rather than autonomous decision-making. The app should classify urgency, reinforce surgeon-approved pathways, and route parents to the right level of action: continue home care, contact the office, request same-day assessment, or seek emergency evaluation [3,6].

Calm guidance for parents

A critical design principle is that the app should foster calm rather than alarm. Parents in the perioperative period do not simply need more information; they need context, normalization, and clear next steps [1,2]. This is especially true in pediatric surgery, where normal recovery findings such as variable appetite, painful swallowing, bruising, irritability, or transient low-grade fever can be frightening when presented without explanation.

A trusted app can reduce this anxiety by using reassurance-first language, procedure-specific timelines, recovery expectations, symptom examples, and explicit urgency tiers [2]. Instead of amplifying uncertainty, it can interrupt the internet spiral by offering immediate, practice-endorsed explanations of what is expected, what should be monitored, and what should trigger escalation.

Benefits to the surgical practice

The expected practice benefits include lower repetitive call burden, improved caregiver adherence, more consistent patient education, earlier identification of complications, and better quality-improvement data [2,4]. App-based pain monitoring at home has also been shown to facilitate pain assessment and empower parents and pediatric patients in postoperative recovery, which is directly relevant to common surgical pathways [5].

**Figure 3:**
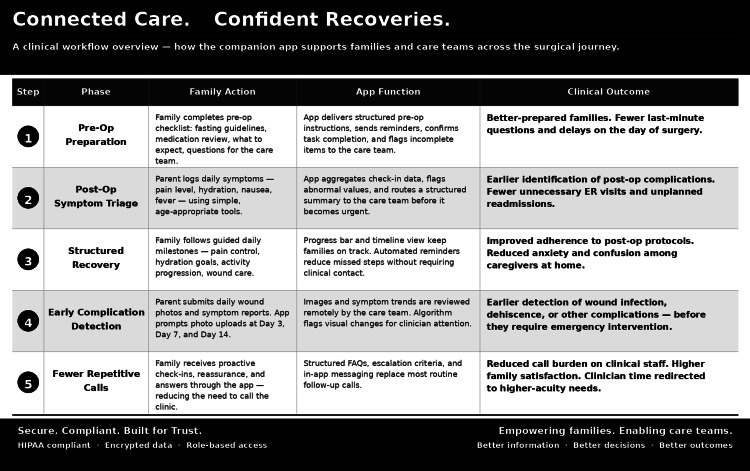
Clinician Dashboard This is an example of a proposed clinician dashboard for structured recovery review and early escalation. This schematic shows how a pediatric surgeon and team member might review a dashboard summary generated from app-reported home recovery data. This schematic was created by the author using Python and the Pillow imaging library (Python Software Foundation; Pillow Contributors, https://python-pillow.org)

Beyond operational efficiency, such a tool can improve the family experience. Parents often judge surgical care not only by the operation itself, but by how supported they feel once the child returns home. A practice-branded companion app may strengthen trust, improve satisfaction, and differentiate the practice as accessible and organized [2,4]. A conceptual clinician dashboard view is shown in Figure 3.

Implementation model

A realistic implementation strategy would begin with a single high-volume outpatient pathway such as tonsillectomy, adenoidectomy, or hernia repair [2]. The first-phase build should include surgeon-approved education, discharge instructions, symptom check-ins, escalation logic, photo upload capability, and recovery summaries. Outcomes could then be measured using call volume, unscheduled visits, caregiver satisfaction, pain-related contacts, hydration concerns, and time to clinical escalation for true complications [2,5].

If successful, the model could expand into neonatal surgery, infant feeding-sensitive procedures, reconstructive pathways, and other specialties where longitudinal home monitoring is important [4]. Integration with existing communication workflows and electronic health record outputs would increase clinical adoption and reduce the risk of creating a parallel system outside the practice.

## Discussion

The value proposition of an AI-driven parent-baby companion app in pediatric surgery lies less in technical novelty than in operational fit. Pediatric surgical care is already organized around standardized instructions, predictable recovery trajectories, and repeated caregiver questions. These are exactly the settings in which structured digital tools can add value [5].

However, implementation should remain conservative. The app should not diagnose complications independently or displace direct clinical judgment. Its role is to improve preparation, standardize guidance, collect structured observations, reduce unnecessary communication friction, and identify situations that warrant clinician review. The most effective versions will likely be procedure-specific, practice-affiliated, and intentionally reassuring in tone [3].

Further study is needed to determine the effect of such platforms on postoperative complications, emergency department utilization, caregiver anxiety, and health care costs in pediatric surgery. Prospective implementation studies within defined surgical pathways would be a practical next step [4].

## Conclusions

A trusted AI-driven parent-baby companion app represents a promising, though as yet unvalidated, approach to extending perioperative support into the home for pediatric surgical families. Based on the available literature in adjacent digital health domains and established principles of caregiver-centered care, such a platform may offer potential benefits by improving caregiver preparedness, reducing repetitive postoperative calls, structuring home recovery data, and supporting earlier recognition of complications, though these benefits remain to be demonstrated prospectively within pediatric surgical populations. The strongest conceptual model is one in which the app reinforces surgeon-directed care, provides calm rather than alarming guidance, and integrates into the existing workflow of the surgical team.

In pediatric surgery, where families assume frontline caregiving responsibilities immediately after discharge, a well-designed companion app could plausibly improve caregiver experience, operational efficiency, and, if rigorously studied, clinical outcomes. Formal prospective evaluation within defined surgical pathways is needed before conclusions about efficacy, safety, or impact on healthcare utilization can be drawn. Future work should prioritize implementation studies in high-volume outpatient procedures, with outcomes including call volume reduction, unscheduled visit rates, caregiver anxiety scores, and time to recognition of true complications.
